# Robotic versus laparoscopic distal pancreatectomy on perioperative outcomes: a systematic review and meta-analysis

**DOI:** 10.1007/s13304-022-01413-3

**Published:** 2022-11-15

**Authors:** Pengyu Li, Hanyu Zhang, Lixin Chen, Tiantong Liu, Menghua Dai

**Affiliations:** 1 Department of General Surgery, Peking Union Medical College Hospital (PUMCH), Peking Union Medical College and Chinese Academy of Medical Sciences, Beijing, China; 2 School of Medicine, Tsinghua University, Beijing, China

**Keywords:** Robotic surgery, Laparoscopic surgery, Distal pancreatectomy, Perioperative outcome

## Abstract

Robotic surgery has become a promising surgical method in minimally invasive pancreatic surgery due to its three-dimensional visualization, tremor filtration, motion scaling, and better ergonomics. Numerous studies have explored the benefits of RDP over LDP in terms of perioperative safety and feasibility, but no consensus has been achieved yet. This article aimed to evaluate the benefits and drawbacks of RDP and LDP for perioperative outcomes. By June 2022, all studies comparing RDP to LDP in the PubMed, the Embase, and the Cochrane Library database were systematically reviewed. According to the heterogeneity, fix or random-effects models were used for the meta-analysis of perioperative outcomes. Odds ratio (OR), weighted mean differences (WMD), and 95% confidence intervals (CI) were calculated. A sensitivity analysis was performed to explore potential sources of high heterogeneity and a trim and fill analysis was used to evaluate the impact of publication bias on the pooled results. Thirty-four studies met the inclusion criteria. RDP provides greater benefit than LDP for higher spleen preservation (OR 3.52 95% CI 2.62–4.73, *p* < 0.0001) and Kimura method (OR 1.93, 95% CI 1.42–2.62, *p* < 0.0001) in benign and low-grade malignant tumors. RDP is associated with lower conversion to laparotomy (OR 0.41, 95% CI 0.33–0.52, *p* < 0.00001), and shorter postoperative hospital stay (WMD − 0.57, 95% CI − 0.92 to − 0.21, *p* = 0.002), but it is more costly. In terms of postoperative complications, there was no difference between RDP and LDP except for 30-day mortality (RDP versus LDP, 0.1% versus 1.0%, *p* = 0.03). With the exception of its high cost, RDP appears to outperform LDP on perioperative outcomes and is technologically feasible and safe. High-quality prospective randomized controlled trials are advised for further confirmation as the quality of the evidence now is not high.

**Supplementary Information:**

The online version contains supplementary material available at 10.1007/s13304-022-01413-3.

## Introduction

Distal pancreatectomy is the standard surgical resection procedure for tumours located at the pancreatic body or tail. With the advancement of surgical techniques, minimally invasive distal pancreatectomy (MIDP) comprising laparoscopic distal pancreatectomy (LDP) and robotic distal pancreatectomy (RDP) has steadily increased in popularity. Compared with open distal pancreatectomy (ODP), MIDP is associated with decreased intraoperative blood loss, a higher rate of spleen preservation, and faster postoperative recovery [1–3]. International practice guidelines for minimally invasive pancreatic surgery recommend MIDP over ODP for benign and low-grade malignant tumours (Grade 1B); moreover, MIDP is a feasible, safe and oncologically equivalent technique for pancreatic malignant tumours (Grade 2B) [4].

In recent years, robotic distal pancreatectomy has increasingly been incorporated into surgical practice [5]. The robotic system provides additional advantages over the conventional laparoscopic system, such as high-resolution three-dimensional (3D) visualization, tremor filtration, motion scaling, and better ergonomics [6, 7], with which complex laparoscopic procedures can theoretically be performed well. Although several studies have compared the clinical efficacy of robotic distal pancreatectomy (RDP) with that of laparoscopic distal pancreatectomy (LDP), no unified conclusion has been reached. With the increase in the studies on this issue, it is necessary to update the meta-analysis. Therefore, we conducted a comprehensive literature review and systematically reviewed the relevant literature to further explore the advantages of RDP compared with LDP in terms of surgical safety, short-term efficacy and cost-effectiveness to provide a comprehensive reference for clinical decision-making.

## Methods

### Study design

This study was conducted according to the Preferred Reporting Items for Systematic Reviews and Meta-Analyses (PRISMA) guidelines [8].

### Search strategy

Three major medical databases were consulted in this research: PubMed, Embase, and the Cochrane Library. Search terms were divided into three parts: (1) robotic or robot-assist or Da Vinci, (2) laparoscopic or laparoscopy, and (3) distal pancreatectomy or left-sided pancreatectomy. The literature research was performed on the perioperative outcomes of LDP and RDP. No beginning date limit was set and the literature search was continuously updated until June 30, 2022. Only English-language studies were selected. In addition, manual searches were conducted on the references of retrieved articles to find other matching articles. Prior to the study selection process, duplicate articles were removed.

Our inclusion criteria were as follows: (1) comparison of RDP and LDP among patients who underwent distal pancreatectomy for benign, borderline malignant, or malignant lesions; (2) report on at least one of the perioperative outcomes listed below. Continuous outcomes had to be provided with the mean and standard deviation (SD). The exclusion criteria were as follows: (1) nonoriginal articles, such as abstracts, case reports and reviews; (2) noncomparative studies; (3) articles with unavailable full text; and (4) peri-operative data that were unable to be extracted from the published studies. Two researchers (Pengyu Li and Hanyu Zhang) independently screened articles by their titles and abstracts, and eliminated articles that met any of the exclusion criteria mentioned above. Any disagreements in study inclusion were resolved through discussion or judged by another researcher (Lixin Chen). The process can be seen in the PRISMA flowchart.

### Data extraction and quality assessment

The literature we finally included had no randomized controlled trials (RCTs), and only case–control and cohort studies. Therefore, we used the modified Newcastle–Ottawa scale (NOS) for quality assessment and scoring. Studies with a score ≥ 6 were considered high-quality studies.

The extracted data included the following; (a) First author's name, publication date, study type, country, number of people included, age, sex, body mass index (BMI). (b) Operation time, estimated blood loss, spleen preservation rate, percentage of the Kimura procedure, R0 resection rate of malignant tumours, conversion to laparotomy, and number of lymph nodes harvested. It is worth emphasizing that the spleen preservation rate is the ratio of successful spleen preservation to intended spleen preservation, rather than the ratio of successful spleen preservation to total cases. The data were not included if the researchers in a particular study did not intend to preserve the spleen. In addition, the cases included in the R0 resection rate and the number of lymph nodes harvested were all malignant tumours. (c) Total complications, major complications, clinical pancreatic fistula, delayed gastric emptying, postoperative haemorrhage, reoperation, 30-day mortality, 90-day mortality, postoperative hospital stay, 90-day readmission, total hospitalization costs, and operation costs. According to the International Study Group of Pancreatic Fistula (ISGPF) guidelines, clinical pancreatic fistula was classified as grade B or C [9]. Complications were graded according to the Clavien–Dindo grading system [10]. Major complications referred to complications of grades III–V. Costs were all converted into US dollars ($).

### Statistical analysis

Review Manager (RevMan) version 5.4 and Stata 16.0 were used for data analysis. Continuous variables were evaluated by the weighted mean difference (WMD) with a 95% confidence interval (95% CI), and dichotomous variables were evaluated using the odds ratios (OR) with 95% CI. Heterogeneity was assessed using *X*^2^ and the *I*^2^ index. The fixed-effect model (FEM) and random effect model (REM) were used based on the value of *I*^2^. Low, moderate, and high heterogeneity were considered for levels of *I*^2^ values of 25–49%, 50–74%, and above 75%, respectively [11]. If *I*^2^ was > 50%, we considered it to have significant heterogeneity and a REM was adopted, then, a sensitivity analysis was performed to explore potential sources. *p* < 0.05 was considered statistically significant. Egger’s test was used to assess the publication bias of the included studies [12]. If there was a publication bias, a trim and fill analysis was further used to evaluate the impact of it on the pooled results.

## Results

### Characteristics of the included studies

**Fig. 1:**
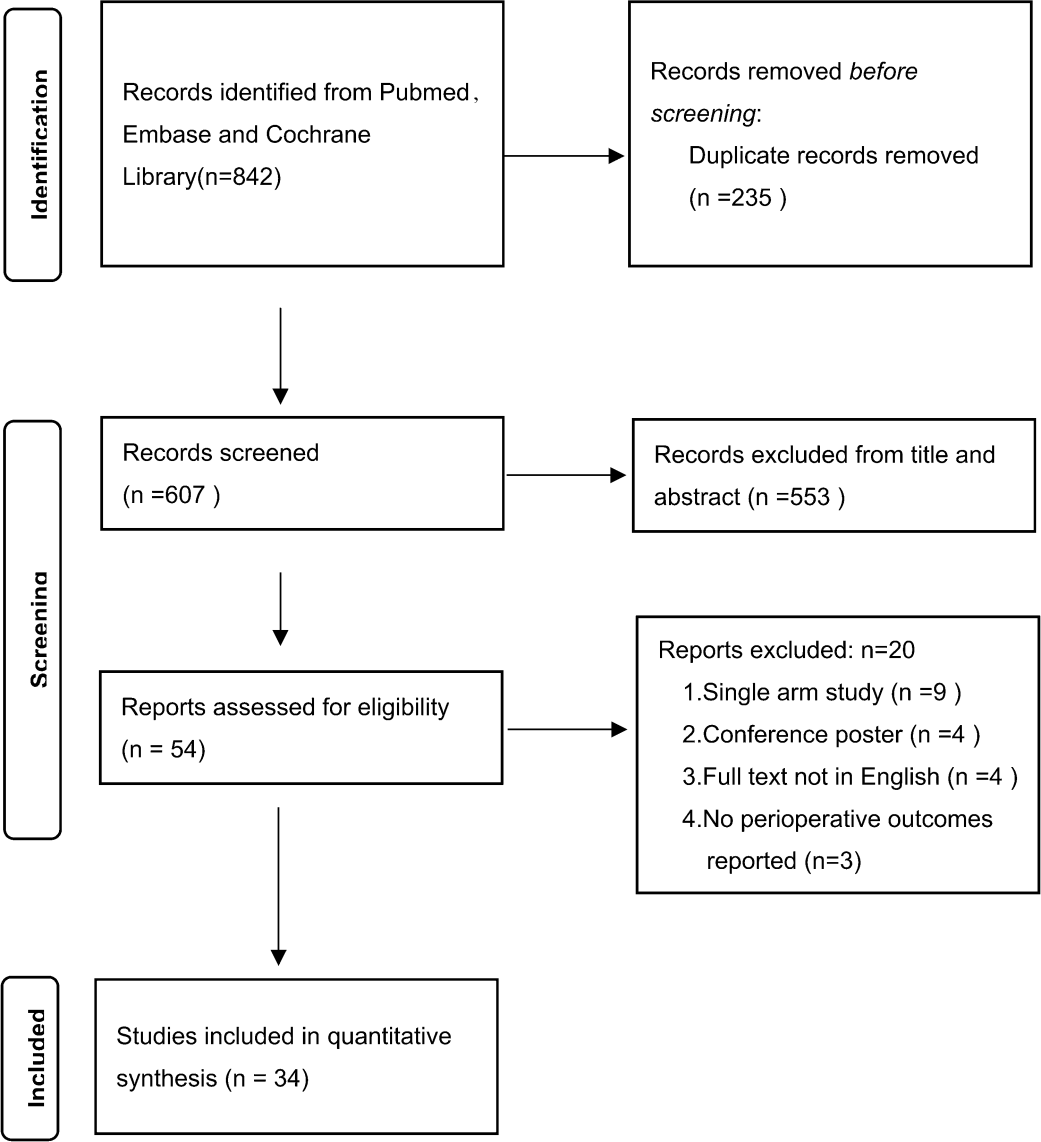
Flowchart of the included studies

A total of 607 studies were retrieved, and 34 relevant studies [13–46] that met the criteria were finally included. Only one study [15] was a prospective nonrandomized study, whereas the others were retrospective studies. The flow diagram of our analysis protocol is shown in Fig. 1. All included studies were of high quality according to the NOS. A total of 5785 patients were included in these studies. There were 2163 patients in the RDP group and 3622 patients in the LDP group. The details of the included literature data are shown in Table 1.

**Table 1:** Characteristics of the included studies

Study	Year	Country	Study design	NOS	Patient number RDP/LDP	Age (year) (mean) RDP/LDP	Male RDP/LDP	BMI (kg/m^2^) (mean) RDP/LDP	ASA III–IV RDP/LDP	Malignant (%) RDP/LDP	Pancreatic stump management (stapler)
Alfieri et al. [13]	2019	Italy	R	8	96/85	NA	46/43	NA	24/18	0%/0%	70.9/69.4%
Benizri et al. [14]	2014	USA	R	7	11/23	50.1/52.3	3/13	25.6/26.5	1/3	0%/13%	0%/52.2%
Butturini et al. [15]	2015	Italy	P	7	22/21	NA	5/6	NA	1/0	13.6%/9.5%	0%/19.0%
Chen et al. [16]	2015	China	R	6	69/50	56.2/56.5	16/29	24.6/24.6	3/2	23.2%/22%	100%/100%
Chen et al. [17]	2022	China	R	7	54/95	50.06/51.74	23/18	24.23/24.23	8/28	14.8%/12.6%	NA
Chopra et al. [18]	2021	USA	R	7	88/17	NA	42/7	NA	76/14	NA	NA
Daouadi et al. [19]	2013	USA	R	7	30/94	59/59	10/33	27.9/29.0	19/51	43.4%/14.95	NA
Duran et al. [20]	2014	Spain	R	8	16/18	61/58.3	9/9	NA	0/3	75%/77.8%	NA
Eckhardt et al. [21]	2016	Germany	R	7	12/29	NA	4/12	NA	NA	0%/6.9%	NA
Goh et al. [22]	2017	Singapore	R	7	8/31	NA	2/18	NA	NA	0%/12.9%	NA
Hong et al. [23]	2020	South Korea	R	8	46/182	51.2/60.2	14/94	24.9、	2/18	26.1%/41.8%	NA
Ielpo et al. [24]	2017	Spain	R	7	28/26	59.7/61.3	16/17	24.1/24.5	3/3	53.6%/50%	NA
Jiang et al. [25]	2020	China	R	7	63/103	44.5/48.8	13/25	22.8/22.6	NA	0%/0%	NA
Kamarajah et al. [26]	2022	UK	R	8	40/47	NA	17/23	27.70/27.91	21/18	60%/51.1%	NA
Kang et al. [27]	2011	South Korea	R	8	20/25	44.5/56.5	8/11	24.2/23.4	NA	0%/0%	NA
Kwon et al. [28]	2021	South Korea	R	7	104/208	50.62/51.23	35/72	24.05/24.06	6/16	23.1%/24.5%	NA
Lai et al. [29]	2015	China	R	8	17/18	61.2/63.2	10/4	24.1/25.7	0/0	23.5%/11.1%	NA
Lee et al. [30]	2014	USA	R	8	27/75	NA	9/36	NA	3/12	14.8%/22.7%	NA
Lee et al. [31]	2020	Singapore	R	6	37/131	58/58	10/57	287./28.2	NA	10.8%/14.5%	NA
Liu et al. [32]	2017	China	R	8	35/35	48.1/49.6	34/47	NA	2/3	74.3%/71.4%	NA
Liu et al. [33]	2018	China	R	8	35/35	58.1/57.8	40/25	24.5/24.1	0/1	100%/100%	NA
Lof et al. [34]	2021	European	R	8	402/402	57/57	165/158	NA	92/87	16.7%/16.7%	36.3%/77.4%
Lyman et al. [35]	2019	USA	R	7	108/139	56.3/59.5	46/75	29.3/29.0	63/83	21.3%/25.2%	NA
Magge et al. [36]	2018	USA	R	7	196/93	62.6/61.3	91/50	29.7/28.2	159/53	67.9%/54.8%	NA
Marino et al. [37]	2020	Spain	R	9	35/35	59.3/58.5	20/19	NA	5/4	60%/57.1%	100%/100%
Najafi et al. [38]	2020	Germany	R	8	24/32	NA	NA	NA	NA	NA	NA
Pastena et al. [39]	2021	Italy	R	7	37/66	50/53	13/20	NA	2/5	10.8%/10.6%	5.4%/45.5%
Raoof et al. [40]	2018	USA	R	7	99/605	NA	45/322	NA	NA	100%/100%	NA
Rodriguez et al. [41]	2018	France	R	7	21/25	NA	6/12	NA	2/5	9.5%/32%	NA
Shin et al. [42]	2022	South Korea	R	9	21/21	62.14/61.33	11/13	23.2/22.8	2/2	100%/100%	100%/100%
Souche et al. [43]	2018	France	R	7	15/23	57/66	3/9	NA	0/0	13.3%/30.4%	100%/100%
Xourafas et al. [44]	2017	USA	R	6	200/694	NA	83/275	NA	135/446	NA	NA
Yang et al. [45]	2020	South Korea	R	6	37/41	42.9/51.3	14/14	23.5/24.1	3/5	NA	NA
Zhang et al. [46]	2017	China	R	7	43/31	47.9/48.7	20/12	23.9/23.3	0/0	18.6%/22.6%	NA

*R* retrospective, *P* prospective, *NA* not available, *RDP* robotic distal pancreatectomy, *LDP* laparoscopic distal pancreatectomy, *BMI* body mass index, *ASA* American Society of Anesthesiologists (ASA) physical status classification system

### Operative outcomes

The operative outcomes of the included studies are described in Table 2.

**Table 2:** Operative outcomes of the included studies

Operative outcomes	Number of studies	Patient numbers	OR/WMD	95% CI	*p* value	*I*^2^ (%)
Operation time	16	2253	15.82	− 2.94, 34.59	0.10	90
Estimated blood loss	7	882	− 58.29	− 82.92, − 33.65	** < 0.001**	26
Intraoperative blood transfusion	19	2799	0.91	0.66, 1.26	0.58	0
Conversion to laparotomy	29	5294	0.41	0.33, 0.52	** < 0.00001**	26
Spleen preservation	12	1181	3.52	2.62, 4.73	** < 0.0001**	20
Kimura procedure	10	764	1.93	1.42, 2.62	** < 0.0001**	61
Number of lymph node dissected	4	178	0.90	− 1.15, 2.96	0.39	0
R0 resection	11	539	1.62	0.76, 3.42	0.21	37

The rate of spleen preservation refers to the proportion of successful spleen preservation in the preoperative intended spleen preservation. Kimura procedure rate refers to the proportion of the Kimura procedure in the spleen preserved surgery. The number of lymph nodes dissected only counts the number of lymph nodes dissected in pancreatic malignancies

Statistically significant differences are given in bold at *p* < 0.05

*OR* odds ratio, *WMD* weighted mean difference

#### Conversion to laparotomy rate

**Fig. 2:**
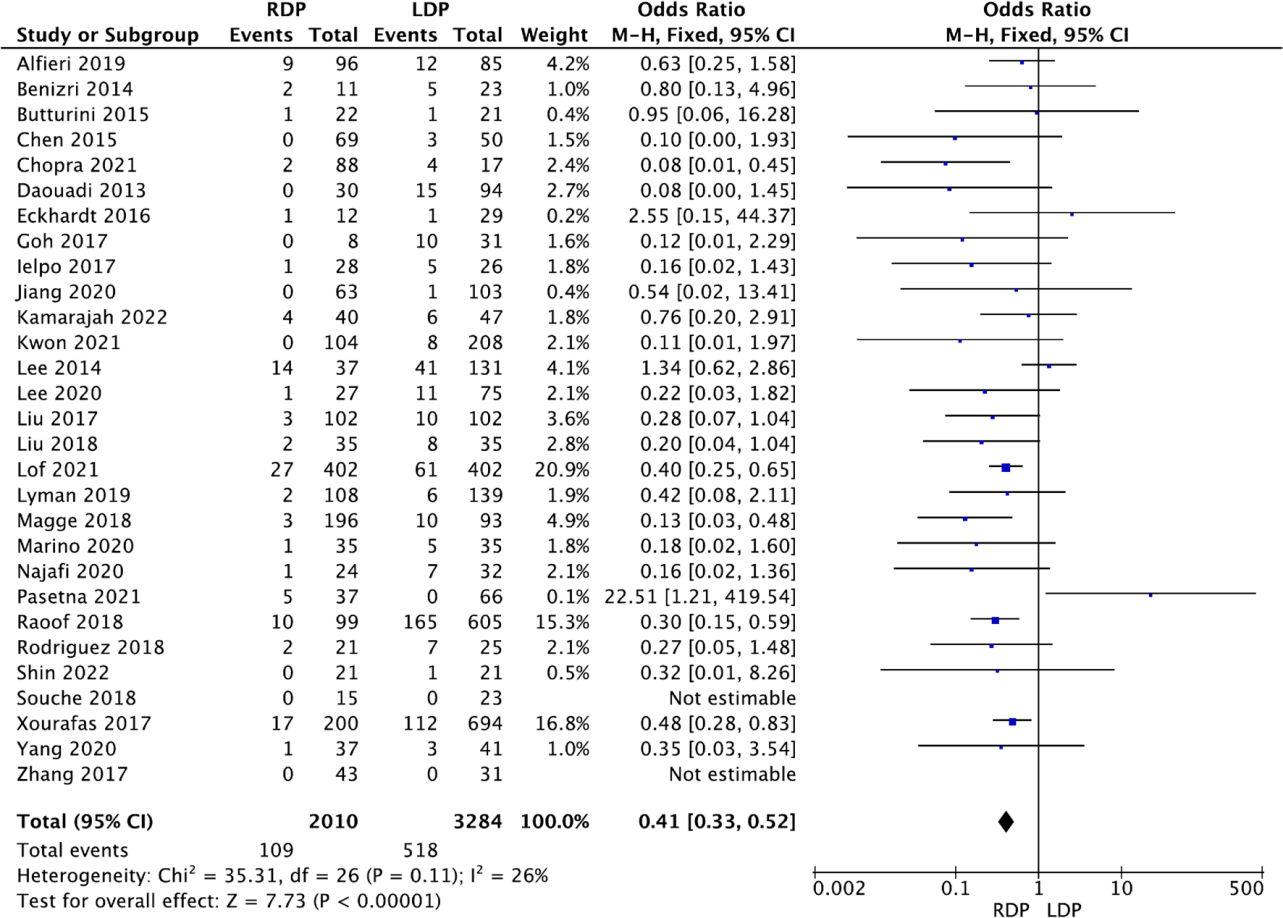
Forest plot showing the meta-analysis of the rate of conversion to laparotomy

A total of 29 studies [13–16, 18, 19, 21, 22, 24–26, 28, 30–46] including 5294 patients reported the conversion rate. The meta-analysis revealed that RDP had a lower conversion rate than LDP (OR 0.41, 95% CI 0.33–0.52, *p* < 0.00001, Fig. 2), with low heterogeneity (*I*^2^ = 26%).

#### Spleen preservation and the Kimura procedure

**Fig. 3:**
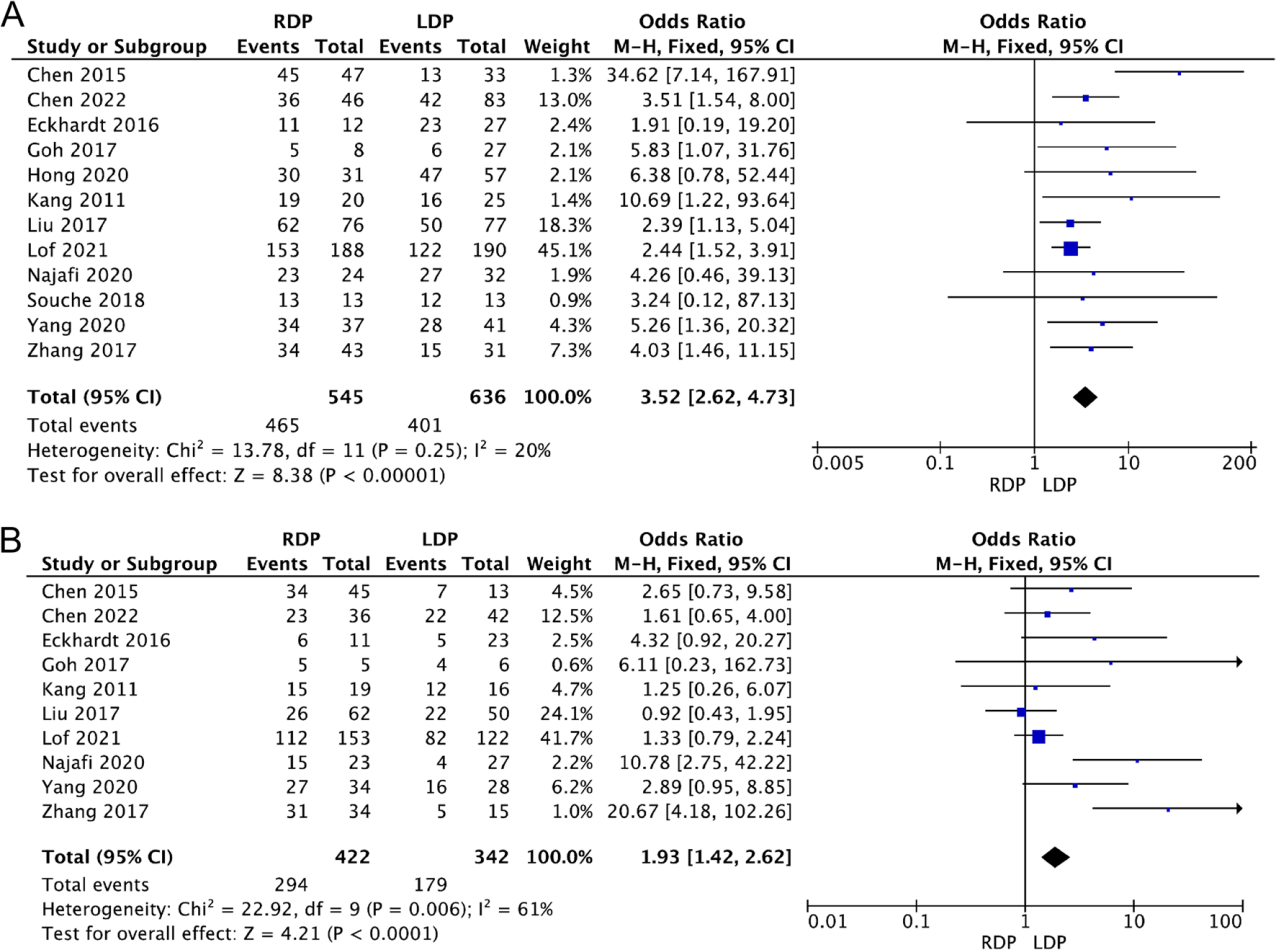
Forest plot displaying the meta-analysis of the spleen preservation rate (**A**) and Kimura procedure rate (**B**)

Twelve studies [16, 17, 21–23, 27, 32, 34, 38, 43, 45, 46] including 1181 patients compared the spleen preservation rate between the RDP and LDP groups. Preservation of the spleen was planned preoperatively for these patients. The included studies had low heterogeneity (*I*^2^ = 20%). The random model results showed that for benign/borderline malignant pancreatic tumours, RDP was associated with a significantly higher spleen preservation rate (OR 3.52 95% CI 2.62–4.73, *p* < 0.0001, Fig. 3A). Ten studies of them [16, 17, 21, 22, 27, 32, 34, 38, 45, 46] reported methods of preserving the spleen. The results showed that RDP was associated with a higher Kimura procedure rate (OR 1.93, 95% CI 1.42–2.62, *p* < 0.0001, Fig. 3B), with moderate heterogeneity (*I*^2^ = 61%).

#### Number of harvested lymph nodes and R0 resection

**Fig. 4:**
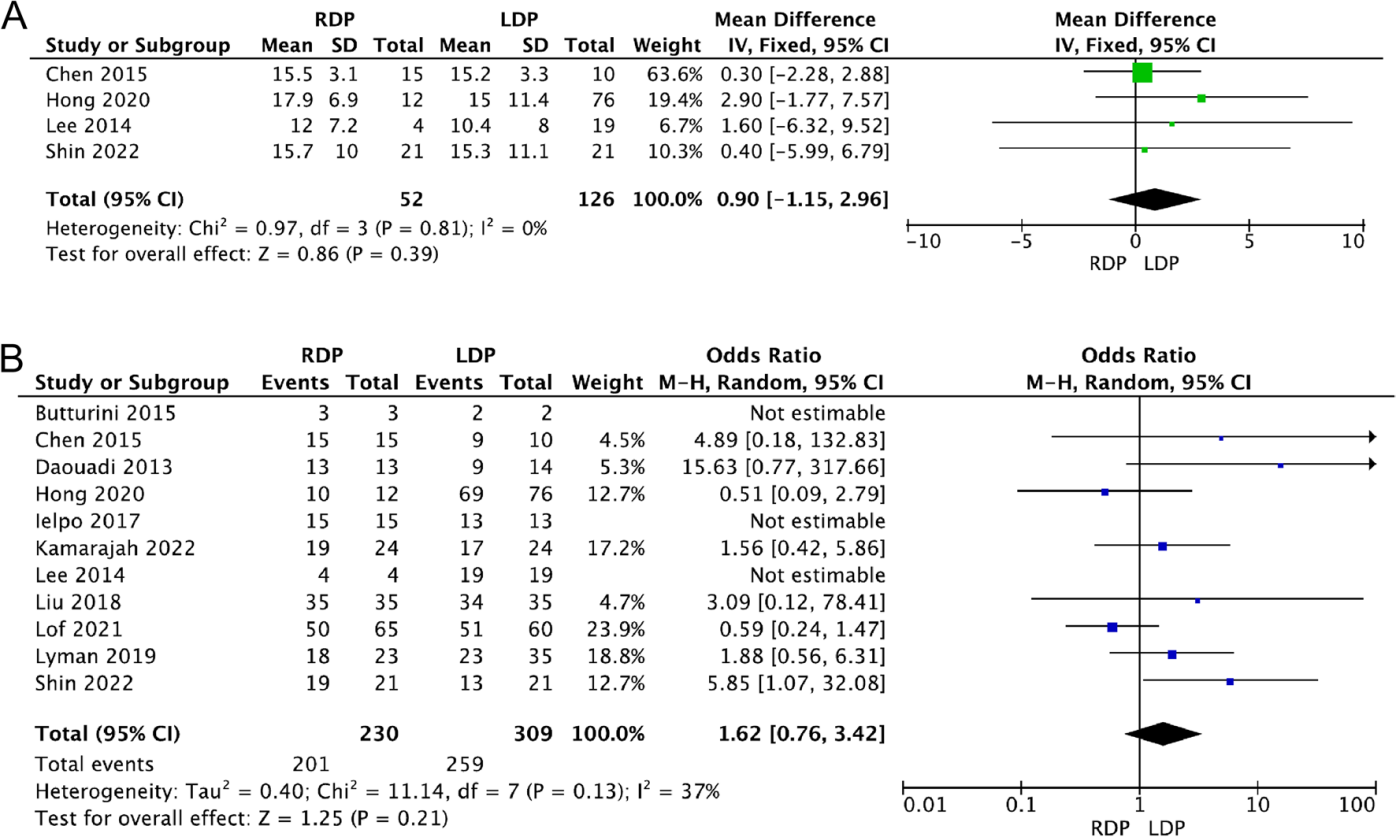
Forest plot displaying the meta-analysis of the number of lymph nodes harvested (**A**) and R0 resection rate (**B**)

Regarding malignant tumours, 4 studies [16, 23, 30, 42] and 11 studies [15, 16, 19, 23, 24, 26, 30, 33–35, 42] reported the number of harvested lymph nodes and R0 resection rate, respectively. There was no heterogeneity in the harvested lymph nodes (*I*^2^=0%), and low heterogeneity in the R0 resection rate (*I*^2^=37%). The results showed that RDP was comparable to LDP in terms of the number of lymph nodes harvested and the R0 resection rate (WMD 0.90, 95% CI − 1.15 to 2.96, *p* = 0.39, Fig. 4A; OR 1.62, 95% CI 0.76–3.42, *p* = 0.21, Fig. 4B). However, only five studies defined R0 as microscopic radical resection of at least 1mm between the tumor at transection or retroperitoneal margin [23, 26, 33–35], while the remaining six studies did not show the definition of R0.

#### Other surgical outcomes

**Fig. 5:**
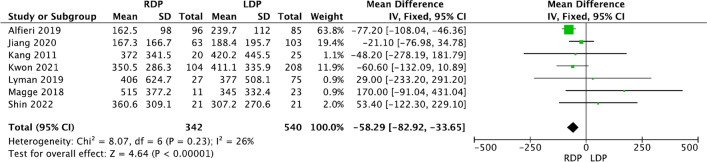
Forest plot showing the meta-analysis on intraoperative estimated blood loss

Seven studies [13, 25, 27, 28, 35, 36, 42] provided detailed data on intraoperative estimated blood loss, respectively. The results showed that RDP led to less intraoperative blood loss (WMD − 58.29, 95% CI − 82.92 to −33.65, *p* < 0.00001, *I*^2^ = 26%, Fig. 5). However, no significant difference between RDP and LDP was found in terms of operation time (S Fig. 1), or blood transfusion (S Fig. 2).

### Postoperative outcomes

The postoperative outcomes of the included studies are described in Table 3.

**Table 3:** Postoperative outcomes of the included studies

Postoperative outcomes	Number of studies	Patient number	OR/WMD	95% CI	*p* value	*I*^2^ (%)
Overall complications	24	2585	0.90	0.75, 1.07	0.22	0
Major complications	23	3424	0.92	0.73, 1.15	0.44	1
Pancreatic fistula (grade B/C)	30	4108	0.91	0.77, 1.08	0.26	0
Delayed gastric emptying	4	1906	1.04	0.54, 2.00	0.91	14
Postoperative hemorrhage	14	2173	0.83	0.52,1.33	0.45	0
Reoperation	23	3996	0.80	0.56, 1.14	0.22	0
Postoperative hospital stay	13	1678	− 0.57	− 0.92, − 0.21	**0.002**	1
30-day mortality	15	3277	0.28	0.09, 0.88	**0.03**	0
90-day mortality	18	3309	0.66	0.31, 1.37	0.26	5
90-day readmission	14	2290	1.03	0.72, 1.47	0.87	27
Total cost	5	729	2910.76	1862.73, 3958.80	** < 0.00001**	86
Operation cost	3	375	2743.40	1011.16, 4475.64	**0.002**	98

Major complications refer to the complications of grade > 2 according to the Clavien–Dindo grade system. The Pancreatic fistula definition is according to the ISGPF criteria

Statistically significant differences are given in bold at *p* < 0.05

#### Clinical pancreatic fistula (grade B/C)

**Fig. 6:**
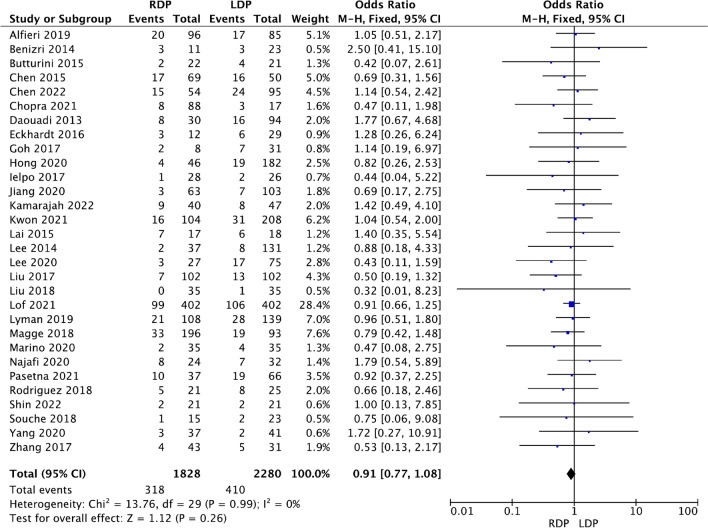
Forest plot showing the meta-analysis of postoperative pancreatic fistula (grade B or C)

Thirty of the included studies [13–19, 21–26, 28–39, 41–43, 45, 46] compared the pancreatic fistula rate between the RDP and LDP groups; however, no difference in the incidence of clinical pancreatic fistulas was observed between the two groups (OR 0.91, 95% CI 0.77–1.08, *p* = 0.26; Fig. 6), with no heterogeneity among the studies (*I*^2^ = 0%).

#### Postoperative hospital stay

**Fig. 7:**
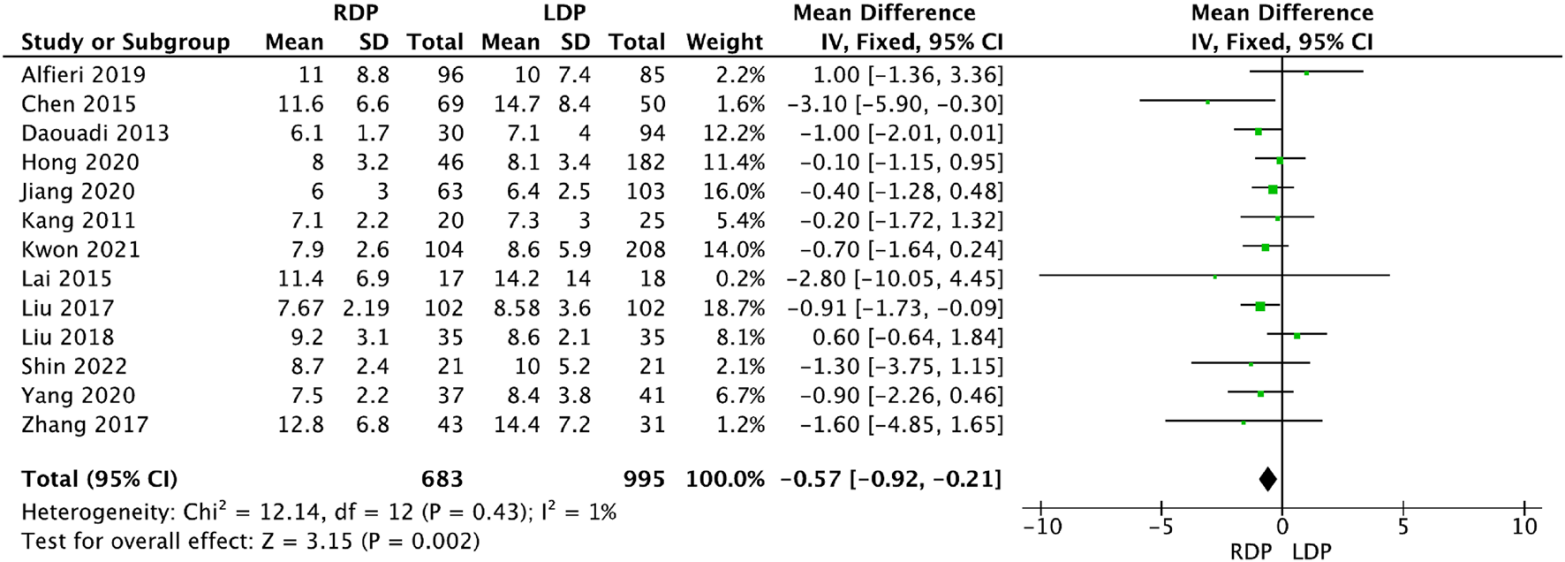
Forest plot displaying the meta-analysis of postoperative hospital stay

Thirty studies [13, 16, 19, 23, 25, 27–29, 32, 33, 42, 45, 46] provided data about the postoperative hospital stay, and the meta-analysis revealed that patients receiving RDP tended to have a shorter postoperative stay than those receiving LDP (WMD − 0.57, 95% CI − 0.92 to − 0.21, *p* = 0.002, Fig. 7), with low heterogeneity (*I*^2^ = 1%).

#### Thirty-day mortality and 90-day mortality

**Fig. 8:**
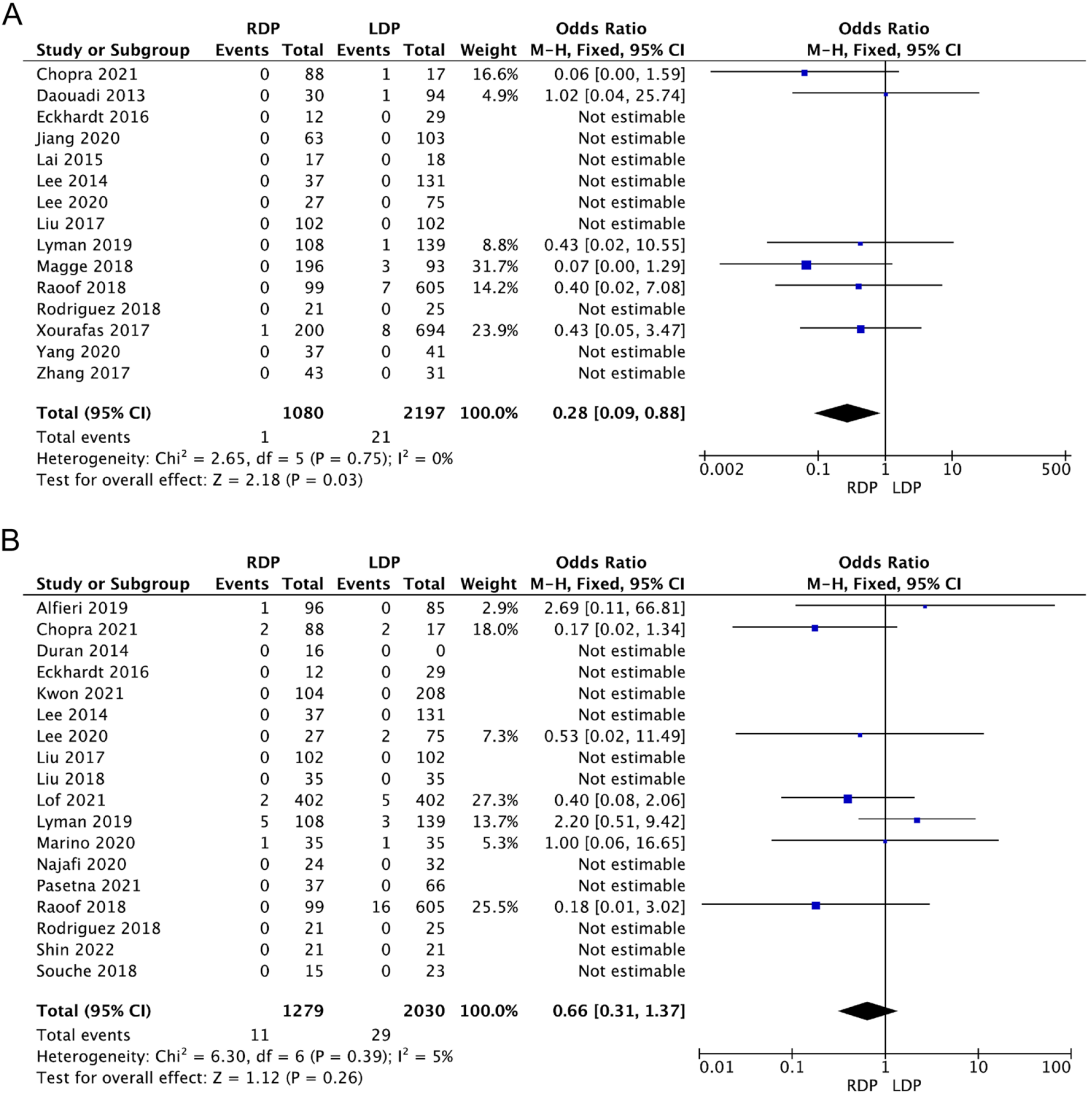
Forest plot showing the meta-analysis of 30-day mortality and 90-day mortality

Fifteen studies [18, 19, 21, 25, 29–32, 35, 36, 40, 41, 44–46] reported 30-day mortality. Meta-analysis indicated that RDP had lower 30-day mortality (OR 0.28, 95% CI 0.09–0.88, *p* = 0.03, Fig. 8A). Notably, 9 studies reported no 30-day mortality in either RDP or LDP. There was no heterogeneity among these 15 studies (*I*^2^ = 0%). In terms of 90-day mortality [13, 18, 20, 21, 28, 30–35, 37–43], there was no difference between the two groups (OR 0.66, 95% CI 0.31–1.37, *p* = 0.26, *I*^2^ = 5%, Fig. 8B).

#### Other complications

Compared with the LDP group, the RDP group had fewer postoperative overall complications (S Fig. 3A), major complications(S Fig. 3B), postoperative haemorrhage(S Fig. 4), and reoperation rates(S Fig. 5), but the differences were not statistically significant. Furthermore, RDP seemed to increase the complications of 90-day readmission (S Fig. 6) and gastric emptying (S Fig. 7), but no statistically significant difference was found.

#### Total cost and operation cost

**Fig. 9:**
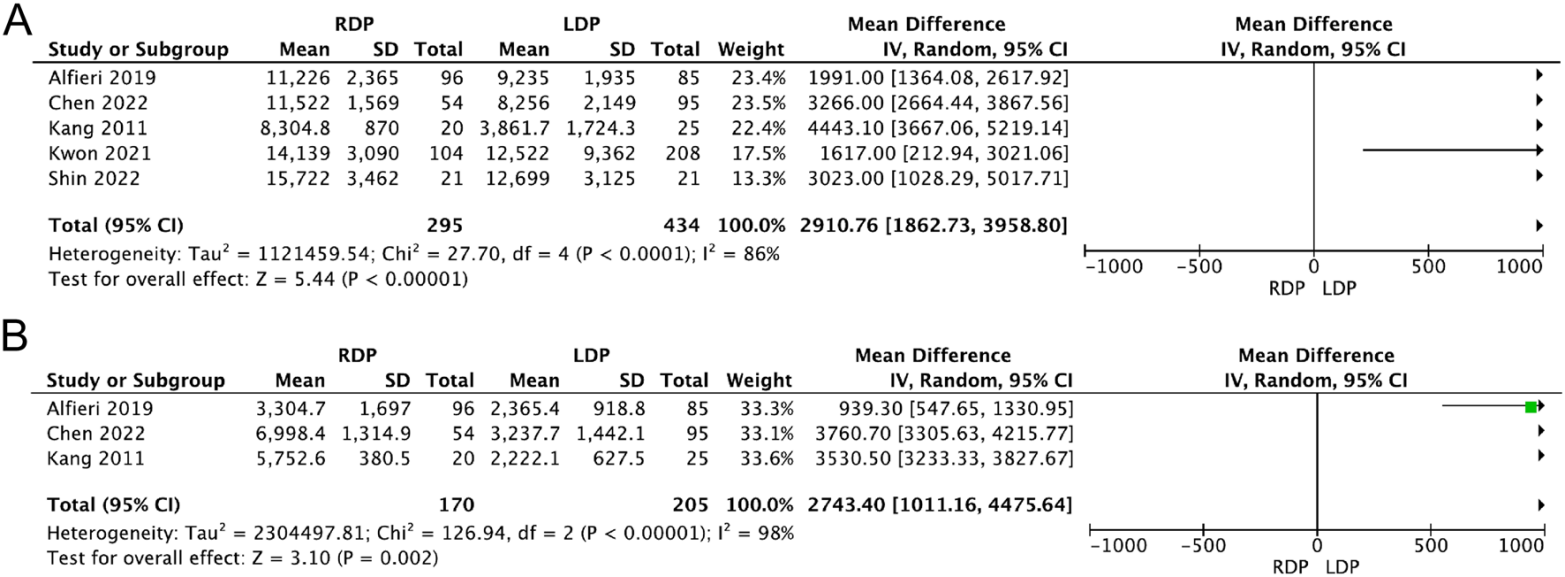
Forest plot displaying the meta-analysis of the total cost (**A**) and operation cost (**B**)

Only five studies [13, 17, 27, 28, 42] and three studies [13, 17, 27] provided complete data about total cost and operation cost, respectively. The results showed that the RDP group was associated with high total cost and operation cost (WMD 2910.76, 95% CI 1862.73–3958.80, *p* < 0.00001, Fig. 9A; WMD 2743.40, 95% CI 1011.16–4475.64, *p* = 0.002, Fig. 9B); however, both of the results had high heterogeneity (*I*^2^ = 86%, *I*^2^ = 98%).

### Sensitivity analysis

We performed a sensitivity analysis on the outcomes of interest with moderate or high heterogeneity to explore their potential sources and assess the robustness of these outcomes. The sensitivity analysis showed that other results were not reversed after sequential removal of each study, except for operation time and operation cost. The *p* value of operation time changed from 0.10 to 0.0004 after excluding the study by Magge et al. [36] and the *p* value of the operation cost changed from 0.04 to 0.22 and 0.10 after excluding the study by Chen et al. [17] and Kang et al. [27], respectively.

### Publication biases

Egger’s tests were performed to assess publication bias. There was no publication bias in any of the outcomes, except the spleen preservation rate and Kimura procedure rate (*p* = 0.000 and *p* = 0.006, respectively) (S Table 1). We further applied a trim and filling analysis to evaluate the impact of publication bias on the results. The analysis showed that the result of spleen-preserving rate was stable, while the result of Kimura procedure rate was inconsistent, indicating a publication bias.

## Discussion

In this meta-analysis, different aspects of the two different minimally invasive approaches to distal pancreatectomy were compared. The results showed that RDP is associated with a higher spleen preservation rate and Kimura method rate in benign and low-grade malignant tumours than LDP. More importantly, RDP is associated with a lower conversion rate to laparotomy, less intraoperative blood loss, shorter postoperative hospital stay and 30-day mortality, although its cost is higher. Overall, RDP is a safe and feasible approach to distal pancreatectomy.

There are no guidelines regarding whether the spleen should be resected in patients with a benign or low-grade malignant pancreatic tumour. Several studies have reported benefits of spleen preservation, such as prevention of overwhelming postsplenectomy infection (OPSI) [47] and cardiovascular complications [48], reduction of intra-abdominal abscess [49] and clinically relevant pancreatic fistula [50]. Different from several previous meta-analyses [13, 51, 52], the spleen preservation rate in our study was the ratio of successful spleen preservation to the planned spleen preservation before surgery, rather than the ratio of successful spleen preservation to the total operations, which can objectively reflect the spleen preservation caused by technical factors. Compared with the meta-analysis by Rompianesi et al. in 2021 [53], the number of studies included in our study was increased and the heterogeneity was low (*I*^2^ = 20%). Our meta-analysis revealed that the rate of RDP in spleen preservation could be 2.52 times higher than that of LDP, showing the advantages of RDP in spleen preservation due to its superior ability to control bleeding from splenic vessels. Although it is worth noting that publication bias existed in our analysis, after using the trim and fill analysis, the result remained significant, indicating the stability of the high spleen preservation of RDP. Nonetheless, the results should be interpreted cautiously. In clinical practice, the Kimura approach is considered the first option to preserve the spleen, with less risk of spleen infarction and left-sided portal hypertension than that with the Warshaw procedure [54, 55]. This meta-analysis revealed a considerable increase in the Kimura procedures performed in RDP. Considering this finding, a robotic approach is indicated for benign and low-grade malignant tumours, where the spleen is to be preserved using the Kimura procedure. However, there was a non-negligible publication bias with respect to the Kimura procedure rate of RDP versus LDP. Therefore, a prospective randomized trial is urgently needed to verify the results.

Conversion to laparotomy, estimated intraoperative blood loss and operation time are important indicators for evaluating minimally invasive surgery. Our results are consistent with previous studies, which revealed that RDP can decrease the conversion rate to laparotomy and estimated intraoperative blood loss. This can be explained by improved instrument dexterity and 3D visualization of the operative field to facilitate the performance of procedures in a narrow operation space and convenience in achieving haemostasis under endoscopy. In addition, another intrinsic benefit of the robot’s two lenses may play an important role. When bleeding contaminates one lens, surgeons can switch to a second ‘eye’ to quickly stop the bleeding, and thereby to avoid laparotomy due to excessive bleeding. There was no significant difference in operation time between RDP and LDP with high heterogeneity in the studies included. There was no mention of whether the operation time included the docking time, whether surgeons performing RDP and LDP were experienced and how difficult the surgery was in both groups in several studies, which gave rise to the unreliable result. A previous systematic review reported that the numbers required to surmount the learning curve are 25.3 (95% CI 22.5–28.3) and 20.7 (95% CI 15.8–26.5) for LDP and RDP, respectively [56]. The number of cases in the RDP group included in this meta-analysis ranged from 8 to 402, and that in the LDP group ranged from 18 to 694. This inevitably incorporates the cases that were in the first phase of the learning curve. More importantly, several studies have reported predictive factors for surgical difficulty in MIDP, including resection line, proximity of the tumour to the major vessel, tumour extension to the peripancreatic tissue, left-sided portal hypertension/splenomegaly and parenchymal thickness at the resection line [57–59], which are likely to increase the operation time and intraoperative blood loss. However, the abovementioned factors in the two groups are not reported in most studies, in which selection bias may exist. Nevertheless, the study by Megga et al. [36] including 196 patients in RDP and 93 patients in LDP showed that the operation time of RDP was statistically lower than that of LDP. Consequently, it can be anticipated that with the proficiency of robotic techniques, the operation time of RDP will be shorter than that of LDP.

In terms of oncologic outcomes, we included studies on malignant tumours, and our results showed that compared with LDP, RDP increased the number of lymph nodes dissected. A previous meta-analysis conducted by Feng et al. [60] concluded that RDP appeared to be associated with a higher R0 resection rate (*p* < 0.0001). However, we considered extracting the data after propensity score matching (PSM) to be more accurate, and the number of relevant studies increased with the year. In our analysis, more studies were included to comprehensively evaluate the impact of RDP on the R0 resection rate. The current meta-analysis revealed that there was no significant difference between the two procedures. Concerning overall survival, we retrieved six studies [18, 23, 32, 34, 40, 42], with a total of 1067 patients with a pathological diagnosis of adenocarcinoma. All studies showed no significant difference in survival between RDP and LDP, indicating the comparability of RDP to LDP. However, margin status is strongly affected by the pathologic evaluation and the definition, and is thus potentially biased by the protocols adopted. In terms of R0 resection rate and prognosis, five studies [23, 26, 33–35] and five studies [18, 23, 32, 34, 42], respectively, showed the definition of R0 (resection margin > 1 mm), while the remaining studies did not show the definition. Therefore, potential bias should also not be neglected and the results should be interpreted cautiously.

Postoperative complications and length of postoperative hospital stay are postoperative indicators reflecting the safety of surgery. Clinical pancreatic fistula, the most common and potentially dangerous complication of DP, may cause lethal haemorrhage and intraperitoneal abscesses [61]. Our meta-analysis showed no significant difference between RDP and LDP with respect to clinical pancreatic fistula (grade B/C). However, a few studies have reported drain management and the pancreas transection plan. As reported in previous studies, early drain removal can reduce clinical pancreatic fistula [62], and a transection plan involving the tail of the pancreas and a use of ultrasonic dissector are risk factors for clinical pancreatic fistula [62–64]. Therefore, comprehensive data are required when comparing the impact of the two approaches on clinical pancreatic fistula. With regard to other postoperative complications, the pooled data showed that the 30-day mortality rate was 0.1% in the RDP group and 1.0% in the LDP group (*p* = 0.03). It should be pointed out that currently the surgical technique is mature and the 30-day mortality is relatively low, hence, several studies claimed no 30-day mortality. Nonetheless, the unique advantages of the robotic approach mentioned above, which allowed for precise intraoperative manipulation and adequate haemostasis, may account for the lower 30-day mortality. In terms of the postoperative hospital stay, RDP reduced the LOS by approximately 0.57 days compared to that after LDP. This may be related to the low conversion rate to laparotomy and reduced trauma in the RDP group. Based on the aforementioned data, RDP appeared more consistent with ERAS (enhanced recovery accelerated surgery).

Hospitalization cost is one of the factors surgeons and patients consider when choosing surgical methods. Our analysis showed that RDP was more costly in terms of hospitalization and operation costs. However, the heterogeneities are too high. Different charging standards could be one cause of the heterogeneity. Although RDP can shorten the length of hospital stay and thus reduce part of the cost, due to the high cost of robots, the total cost and surgical cost are still higher than those of LDP [27, 39]. It is believed that with the continuous development of robotic techniques, costs will decrease, allowing more patients to access superior surgical methods.

Recently, several studies based on the data analysis of multicentre and large-scale studies reported the benchmark values of MIDP to identify the best achievable results and define optimal perioperative outcomes, with the intention of assessing and enhancing the surgery quality [65, 66]. Muller et al. [66] reported that benchmark values of RDP included: operation time ≤ 300 min, estimated blood loss ≤ 150 ml, conversion rate ≤ 3%, major complication rate ≤ 26.7%, clinical pancreatic fistula rate ≤ 32%, lymph node retrieval for PADC ≥ 9, and R0 resection rate for PDAC ≥ 83%. In the majority of the included studies, there was a disparity between the outcomes and the benchmark values. Although RDP has demonstrated its superiority, surgeons must work towards benchmark levels to maximize its benefits.

This meta-analysis summarizes the relevant data of high-quality literature that could be retrieved thus far and reveals the benefits of RDP over LDP. However, the results should be interpreted with caution due to the following limitations. First the included studies were restricted to retrospective or prospective non-randomized controlled studies published in English, which may affect the accuracy of the results. Second, some of the included literature did not provide complete data. A few articles use an algorithm to estimate the mean and standard deviation (SD) of continuous variables [67, 68]. We thought that  this method had certain flaws, and therefore, in our analysis incomplete data were excluded, which may affect the final results. Third, publication bias existed in several outcomes, which impacted the stability of the results. Meanwhile, some studies reported on data obtained during the learning curve stage, which resulted in marked heterogeneity. Ultimately, we look forward to randomized controlled studies to further demonstrate the difference between the robotic and laparoscopic systems in the short and long-term outcomes of distal pancreatectomy.

## Conclusion

This meta-analysis suggested that RDP is comparable to LDP in terms of perioperative outcomes and oncologic outcomes. Current studies proved that the robotic system had superiority in terms of a higher spleen preservation rate and Kimura method rate in patients with benign and low-grade malignant tumours, and more lymph nodes were dissected in cases of malignant tumours. More importantly, RDP is associated with a lower rate conversion to laparotomy, and shorter postoperative hospital stay, but the procedure is more costly. Nonetheless, the evidence grade is low, and large-scale RCTs are needed to further demonstrate the benefits of RDP.

## Supplementary Information

Below is the link to the electronic supplementary material.

S Fig. 1 Forest plot showing the meta-analysis of operation time (TIFF 326 KB)

S Fig. 2 Forest plot showing the meta-analysis of the rate of intraoperative blood transfusion (TIFF 351 KB)

S Fig. 3 Forest plot displaying the meta-analysis of overall complications (A) and major complications (B) (TIFF 814 KB)

S Fig. 4 Forest plot displaying the meta-analysis of postoperative haemorrhage (TIFF 276 KB)

S Fig. 5 Forest plot showing the meta-analysis of reoperation (TIFF 397 KB)

S Fig. 6 Forest plot showing the meta-analysis of 90-day readmission (TIFF 289 KB)

S Fig. 7 Forest plot showing the meta-analysis of delayed gastric emptying (TIFF 151 KB)

Supplementary file8 (DOCX 15 KB)

## Data Availability

The data that support the findings of the meta-analysis are available within the article and its supplementary information files.

## References

[1] de Rooij T, van Hilst J, van Santvoort H, Boerma D, van den Boezem P, Daams F, van Dam R, Dejong C, van Duyn E, Dijkgraaf M (2019). Minimally invasive versus open distal pancreatectomy (LEOPARD): a multicenter patient-blinded randomized controlled trial. Ann Surg.

[2] Raghupathy J, Lee C-Y, Huan SKW, Koh Y-X, Tan E-K, Teo J-Y, Cheow P-C, Ooi LLPJ, Chung AYF, Chan C-Y, Goh BKP (2022). Propensity-score matched analyses comparing clinical outcomes of minimally invasive versus open distal pancreatectomies: a single-center experience. World J Surg.

[3] Korrel M, Vissers FL, van Hilst J, de Rooij T, Dijkgraaf MG, Festen S, Groot Koerkamp B, Busch OR, Luyer MD, Sandström P, Abu Hilal M, Besselink MG, Björnsson B (2021). Minimally invasive versus open distal pancreatectomy: an individual patient data meta-analysis of two randomized controlled trials. HPB (Oxford).

[4] Asbun HJ, Moekotte AL, Vissers FL (2020). The miami international evidence-based guidelines on minimally invasive pancreas resection. Ann Surg.

[5] Levi Sandri GB, Abu Hilal M, Dokmak S, Edwin B, Hackert T, Keck T, Khatkov I, Besselink MG, Boggi U (2022). Figures do matter: a literature review on 4,587 robotic pancreatic resections and their implications on training. J Hepatobiliary Pancreat Sci.

[6] Peters BS, Armijo PR, Krause C, Choudhury SA, Oleynikov D (2018). Review of emerging surgical robotic technology. Surg Endosc.

[7] Zwart MJW, Jones LR, Fuente I (2021). Performance with robotic surgery versus 3D- and 2D-laparoscopy during pancreatic and biliary anastomoses in a biotissue model: pooled analysis of two randomized trials. Surg Endosc.

[8] Moher D, Liberati A, Tetzlaff J, Altman DG (2009). Preferred reporting items for systematic reviews and meta-analyses: the PRISMA statement. PLoS Med.

[9] Bassi C, Marchegiani G, Dervenis C (2017). The 2016 update of the International Study Group (ISGPS) definition and grading of postoperative pancreatic fistula: 11 years after. Surgery.

[10] Dindo D, Demartines N, Clavien P-A (2004). Classification of surgical complications: a new proposal with evaluation in a cohort of 6336 patients and results of a survey. Ann Surg.

[11] Higgins JPT, Thompson SG, Deeks JJ, Altman DG (2003). Measuring inconsistency in meta-analyses. BMJ.

[12] Egger M, Davey Smith G, Schneider M, Minder C (1997). Bias in meta-analysis detected by a simple, graphical test. BMJ.

[13] Alfieri S, Butturini G, Boggi U, Pietrabissa A, Morelli L, Vistoli F, Damoli I, Peri A, Fiorillo C, Pugliese L, Ramera M, De Lio N, Di Franco G, Esposito A, Landoni L, Rosa F, Menghi R, Doglietto GB, Quero G (2019). Short-term and long-term outcomes after robot-assisted versus laparoscopic distal pancreatectomy for pancreatic neuroendocrine tumors (pNETs): a multicenter comparative study. Langenbeck's Arch Surg.

[14] Benizri EI, Germain A, Ayav A, Bernard JL, Zarnegar R, Benchimol D, Bresler L, Brunaud L (2014). Short-term perioperative outcomes after robot-assisted and laparoscopic distal pancreatectomy. J Robot Surg.

[15] Butturini G, Damoli I, Crepaz L, Malleo G, Marchegiani G, Daskalaki D, Esposito A, Cingarlini S, Salvia R, Bassi C (2015). A prospective non-randomised single-center study comparing laparoscopic and robotic distal pancreatectomy. Surg Endosc.

[16] Chen S, Zhan Q, Chen JZ, Jin JB, Deng XX, Chen H, Shen BY, Peng CH, Li HW (2015). Robotic approach improves spleen-preserving rate and shortens postoperative hospital stay of laparoscopic distal pancreatectomy: a matched cohort study. Surg Endosc.

[17] Chen P, Zhou B, Wang T, Hu X, Ye Y, Guo W (2022). Comparative efficacy of robot-assisted and laparoscopic distal pancreatectomy: a single-center comparative study. J Healthc Eng.

[18] Chopra A, Nassour I, Zureikat A, Paniccia A (2021). Perioperative and oncologic outcomes of open, laparoscopic, and robotic distal pancreatectomy for pancreatic adenocarcinoma. Updates Surg.

[19] Daouadi M, Zureikat AH, Zenati MS, Choudry H, Tsung A, Bartlett DL, Hughes SJ, Lee KK, Moser AJ, Zeh HJ (2013). Robot-assisted minimally invasive distal pancreatectomy is superior to the laparoscopic technique. Ann Surg.

[20] Duran H, Ielpo B, Caruso R, Ferri V, Quijano Y, Diaz E, Fabra I, Oliva C, Olivares S, Vicente E (2014). Does robotic distal pancreatectomy surgery offer similar results as laparoscopic and open approach? A comparative study from a single medical center. Int J Med Robot.

[21] Eckhardt S, Schicker C, Maurer E, Fendrich V, Bartsch DK (2016). Robotic-assisted approach improves vessel preservation in spleen-preserving distal pancreatectomy. Dig Surg.

[22] Goh BKP, Chan CY, Soh HL, Lee SY, Cheow PC, Chow PKH, Ooi LLPJ, Chung AYF (2017). A comparison between robotic-assisted laparoscopic distal pancreatectomy versus laparoscopic distal pancreatectomy. Int J Med Robot Comput Assist Surg.

[23] Hong S, Song KB, Madkhali AA, Hwang K, Yoo D, Lee JW, Youn WY, Alshammary S, Park Y, Lee W, Kwon J, Lee JH, Hwang DW, Kim SC (2020). Robotic versus laparoscopic distal pancreatectomy for left-sided pancreatic tumors: a single surgeon's experience of 228 consecutive cases. Surg Endosc.

[24] Ielpo B, Duran H, Diaz E, Fabra I, Caruso R, Malavé L, Ferri V, Nuñez J, Ruiz-Ocaña A, Jorge E, Lazzaro S, Kalivaci D, Quijano Y, Vicente E (2017). Robotic versus laparoscopic distal pancreatectomy: a comparative study of clinical outcomes and costs analysis. Int J Surg.

[25] Jiang Y, Zheng K, Zhang S, Shao Z, Cheng P, Zhang Y, Jin G, He T (2020). Robot-assisted distal pancreatectomy improves spleen preservation rate versus laparoscopic distal pancreatectomy for benign and low-grade malignant lesions of the pancreas. Transl Cancer Res.

[26] Kamarajah S, Sutandi N, Sen G, Hammond J, Manas D, French J, White S (2022). Comparative analysis of open, laparoscopic and robotic distal pancreatic resection: the United Kingdom′s first single-centre experience. J Minimal Access Surg.

[27] Kang CM, Kim DH, Lee WJ, Chi HS (2011). Conventional laparoscopic and robot-assisted spleen-preserving pancreatectomy: does da Vinci have clinical advantages?. Surg Endosc.

[28] Kwon J, Lee JH, Park SY, Park Y, Lee W, Song KB, Hwang DW, Kim SC (2022). A comparison of robotic versus laparoscopic distal pancreatectomy: propensity score matching analysis. Int J Med Robot.

[29] Lai EC, Tang CN (2015). Robotic distal pancreatectomy versus conventional laparoscopic distal pancreatectomy: a comparative study for short-term outcomes. Front Med.

[30] Lee SY, Allen PJ, Sadot E, D'Angelica MI, DeMatteo RP, Fong Y, Jarnagin WR, Kingham TP (2015). Distal pancreatectomy: a single institution's experience in open, laparoscopic, and robotic approaches. J Am Coll Surg.

[31] Lee SQ, Kabir T, Koh YX, Teo JY, Lee SY, Kam JH, Cheow PC, Jeyaraj PR, Chow PKH, Ooi LL, Chung AYF, Chan CY, Goh BKP (2020). A single institution experience with robotic and laparoscopic distal pancreatectomies. Ann Hepatobiliary Pancreat Surg.

[32] Liu R, Liu Q, Zhao ZM, Tan XL, Gao YX, Zhao GD (2017). Robotic versus laparoscopic distal pancreatectomy: a propensity score-matched study. J Surg Oncol.

[33] Qu L, Zhiming Z, Xianglong T, Yuanxing G, Yong X, Rong L, Yee LW (2018). Short- and mid-term outcomes of robotic versus laparoscopic distal pancreatosplenectomy for pancreatic ductal adenocarcinoma: a retrospective propensity score-matched study. Int J Surg.

[34] Lof S, van der Heijde N, Abuawwad M (2021). Robotic versus laparoscopic distal pancreatectomy: multicentre analysis. Br J Surg.

[35] Lyman WB, Passeri M, Sastry A, Cochran A, Iannitti DA, Vrochides D, Baker EH, Martinie JB (2019). Robotic-assisted versus laparoscopic left pancreatectomy at a high-volume, minimally invasive center. Surg Endosc.

[36] Magge DR, Zenati MS, Hamad A, Rieser C, Zureikat AH, Zeh HJ, Hogg ME (2018). Comprehensive comparative analysis of cost-effectiveness and perioperative outcomes between open, laparoscopic, and robotic distal pancreatectomy. HPB (Oxford).

[37] Marino MV, Mirabella A, Gomez Ruiz M, Komorowski AL (2020). Robotic-assisted versus laparoscopic distal pancreatectomy: the results of a case-matched analysis from a tertiary care center. Dig Surg.

[38] Najafi N, Mintziras I, Wiese D, Albers MB, Maurer E, Bartsch DK (2020). A retrospective comparison of robotic versus laparoscopic distal resection and enucleation for potentially benign pancreatic neoplasms. Surg Today.

[39] De Pastena M, Esposito A, Paiella S, Surci N, Montagnini G, Marchegiani G, Malleo G, Secchettin E, Casetti L, Ricci C, Landoni L, Bovo C, Bassi C, Salvia R (2021). Cost-effectiveness and quality of life analysis of laparoscopic and robotic distal pancreatectomy: a propensity score-matched study. Surg Endosc.

[40] Raoof M, Nota CLMA, Melstrom LG, Warner SG, Woo Y, Singh G, Fong Y (2018). Oncologic outcomes after robot-assisted versus laparoscopic distal pancreatectomy: analysis of the National Cancer Database. J Surg Oncol.

[41] Rodriguez M, Memeo R, Leon P, Panaro F, Tzedakis S, Perotto O, Varatharajah S, de Angelis N, Riva P, Mutter D, Navarro F, Marescaux J, Pessaux P (2018). Which method of distal pancreatectomy is cost-effective among open, laparoscopic, or robotic surgery?. Hepatobiliary Surg Nutr.

[42] Shin D, Kwon J, Lee JH, Park SY, Park Y, Lee W, Song KB, Hwang DW, Kim SC (2022). Robotic versus laparoscopic distal pancreatectomy for pancreatic ductal adenocarcinoma: a propensity score-matched analysis. Hepatobiliary Pancreat Dis Int.

[43] Souche R, Herrero A, Bourel G, Chauvat J, Pirlet I, Guillon F, Nocca D, Borie F, Mercier G, Fabre JM (2018). Robotic versus laparoscopic distal pancreatectomy: a French prospective single-center experience and cost-effectiveness analysis. Surg Endosc.

[44] Xourafas D, Ashley SW, Clancy TE (2017). Comparison of perioperative outcomes between open, laparoscopic, and robotic distal pancreatectomy: an analysis of 1815 patients from the ACS-NSQIP procedure-targeted pancreatectomy database. J Gastrointest Surg.

[45] Yang SJ, Hwang HK, Kang CM, Lee WJ (2020). Revisiting the potential advantage of robotic surgical system in spleen-preserving distal pancreatectomy over conventional laparoscopic approach. Ann Transl Med.

[46] Zhang J, Jin J, Chen S, Gu J, Zhu Y, Qin K, Zhan Q, Cheng D, Chen H, Deng X, Shen B, Peng C (2017). Minimally invasive distal pancreatectomy for PNETs: laparoscopic or robotic approach?. Oncotarget.

[47] Sinwar PD (2014). Overwhelming post splenectomy infection syndrome—review study. Int J Surg.

[48] Weledji EP (2014). Benefits and risks of splenectomy. Int J Surg.

[49] Lee W, Hwang DW, Han H-S (2022). Comparison of infectious complications after spleen preservation versus splenectomy during laparoscopic distal pancreatectomy for benign or low-grade malignant pancreatic tumors: a multicenter, propensity score-matched analysis. J Hepatobiliary Pancreat Sci.

[50] Șandra-Petrescu F, Tzatzarakis E, Mansour Basha M, Rückert F, Reissfelder C, Birgin E, Rahbari NN (2022). Impact of spleen preservation on the incidence of postoperative pancreatic fistula after distal pancreatectomy: is less more?. Pancreatology.

[51] Kamarajah SK, Sutandi N, Robinson SR, French JJ, White SA (2019). Robotic versus conventional laparoscopic distal pancreatic resection: a systematic review and meta-analysis. HPB (Oxford).

[52] Hu Y-H, Qin Y-F, Yu D-D, Li X, Zhao Y-M, Kong D-J, Jin W, Wang H (2020). Meta-analysis of short-term outcomes comparing robot-assisted and laparoscopic distal pancreatectomy. J Comp Eff Res.

[53] Rompianesi G, Montalti R, Ambrosio L, Troisi RI (2021). Robotic versus laparoscopic surgery for spleen-preserving distal pancreatectomies: systematic review and meta-analysis. J Pers Med.

[54] Yu X, Li H, Jin C, Fu D, Di Y, Hao S, Li J (2015). Splenic vessel preservation versus Warshaw's technique during spleen-preserving distal pancreatectomy: a meta-analysis and systematic review. Langenbecks Arch Surg.

[55] Ferrone CR, Konstantinidis IT, Sahani DV, Wargo JA, Fernandez-del Castillo C, Warshaw AL (2011). Twenty-three years of the Warshaw operation for distal pancreatectomy with preservation of the spleen. Ann Surg.

[56] Chan KS, Wang ZK, Syn N, Goh BKP (2021). Learning curve of laparoscopic and robotic pancreas resections: a systematic review. Surgery.

[57] Deiro G, De Pastena M, Paiella S, Balduzzi A, Montagnini G, Andreotti E, Casetti L, Landoni L, Salvia R, Esposito A (2021). Assessment of difficulty in laparoscopic distal pancreatectomy: a modification of the Japanese difficulty scoring system—a single-center high-volume experience. J Hepatobiliary Pancreat Sci.

[58] Ohtsuka T, Ban D, Nakamura Y (2018). Difficulty scoring system in laparoscopic distal pancreatectomy. J Hepatobiliary Pancreat Sci.

[59] Partelli S, Ricci C, Rancoita PMV, Montorsi R, Andreasi V, Ingaldi C, Arru G, Pecorelli N, Crippa S, Alberici L, Di Serio C, Casadei R, Falconi M (2020). Preoperative predictive factors of laparoscopic distal pancreatectomy difficulty. HPB (Oxford).

[60] Feng Q, Jiang C, Feng X, Du Y, Liao W, Jin H, Liao M, Zeng Y, Huang J (2021). Robotic versus laparoscopic distal pancreatectomy for pancreatic ductal adenocarcinoma: a systematic review and meta-analysis. Front Oncol.

[61] Kawaida H, Kono H, Hosomura N, Amemiya H, Itakura J, Fujii H, Ichikawa D (2019). Surgical techniques and postoperative management to prevent postoperative pancreatic fistula after pancreatic surgery. World J Gastroenterol.

[62] Seykora TF, Liu JB, Maggino L, Pitt HA, Vollmer CM (2020). Drain management following distal pancreatectomy: characterization of contemporary practice and impact of early removal. Ann Surg.

[63] Qian L, Hu B, Wang J, Lu X, Deng X, Chai W, Xu Z, Wang W, Shen B (2022). Impact of the transection plan on postoperative pancreatic fistulas occurring after robot-assisted distal pancreatectomy for nonmalignant pancreatic neoplasms. Surg Endosc.

[64] Pulvirenti A, Landoni L, Borin A, De Pastena M, Fontana M, Pea A, Esposito A, Casetti L, Tuveri M, Paiella S, Marchegiani G, Malleo G, Salvia R, Bassi C (2019). Reinforced stapler versus ultrasonic dissector for pancreatic transection and stump closure for distal pancreatectomy: a propensity matched analysis. Surgery.

[65] Giani A, van Ramshorst T, Mazzola M (2022). Benchmarking of minimally invasive distal pancreatectomy with splenectomy: European multicentre study. Br J Surg.

[66] Müller PC, Breuer E, Nickel F (2022). Robotic distal pancreatectomy, a novel standard of care? Benchmark values for surgical outcomes from 16 international expert centers. Ann Surg.

[67] Wan X, Wang W, Liu J, Tong T (2014). Estimating the sample mean and standard deviation from the sample size, median, range and/or interquartile range. BMC Med Res Methodol.

[68] Luo D, Wan X, Liu J, Tong T (2018). Optimally estimating the sample mean from the sample size, median, mid-range, and/or mid-quartile range. Stat Methods Med Res.

