# Supplementary figures and images for: Robotic versus laparoscopic distal pancreatectomy on perioperative outcomes: a systematic review and meta-analysis
Source: Updates Surg. 2022 Nov 15;75(1):7–21. doi: 10.1007/s13304-022-01413-3 (PMC9834369; doi:10.1007/s13304-022-01413-3)

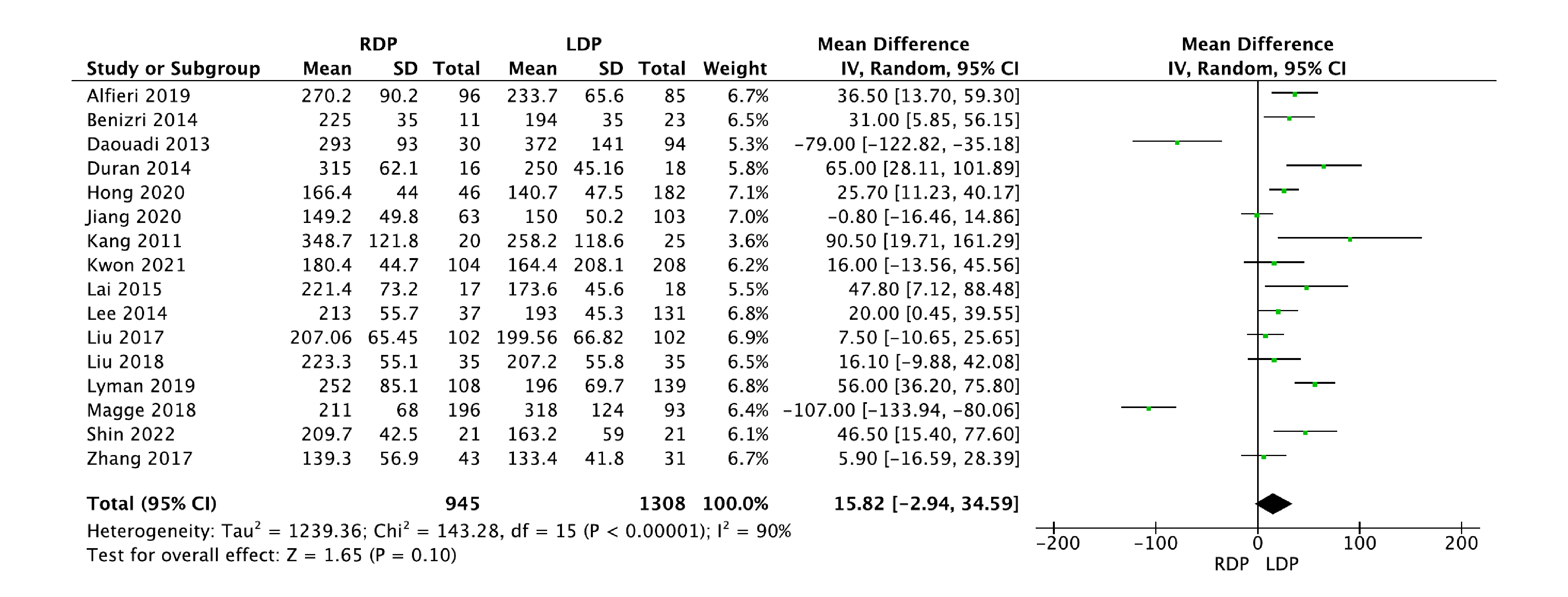

Supplement: Supplementary file 1 — S Fig. 1 Forest plot showing the meta-analysis of operation time (TIFF 326 KB) [file 13304_2022_1413_MOESM1_ESM.tiff]

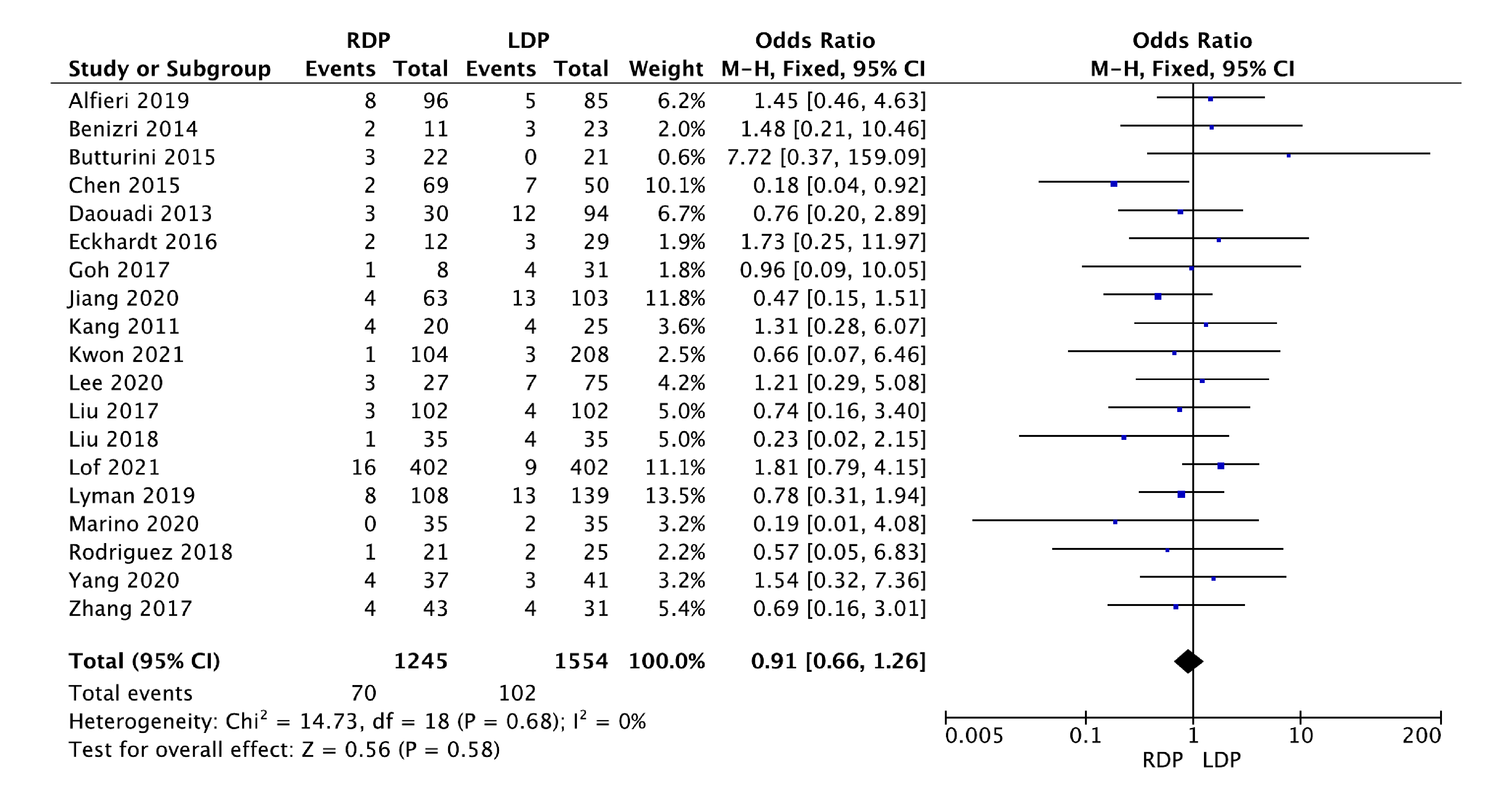

Supplement: Supplementary file 2 — S Fig. 2 Forest plot showing the meta-analysis of the rate of intraoperative blood transfusion (TIFF 351 KB) [file 13304_2022_1413_MOESM2_ESM.tiff]

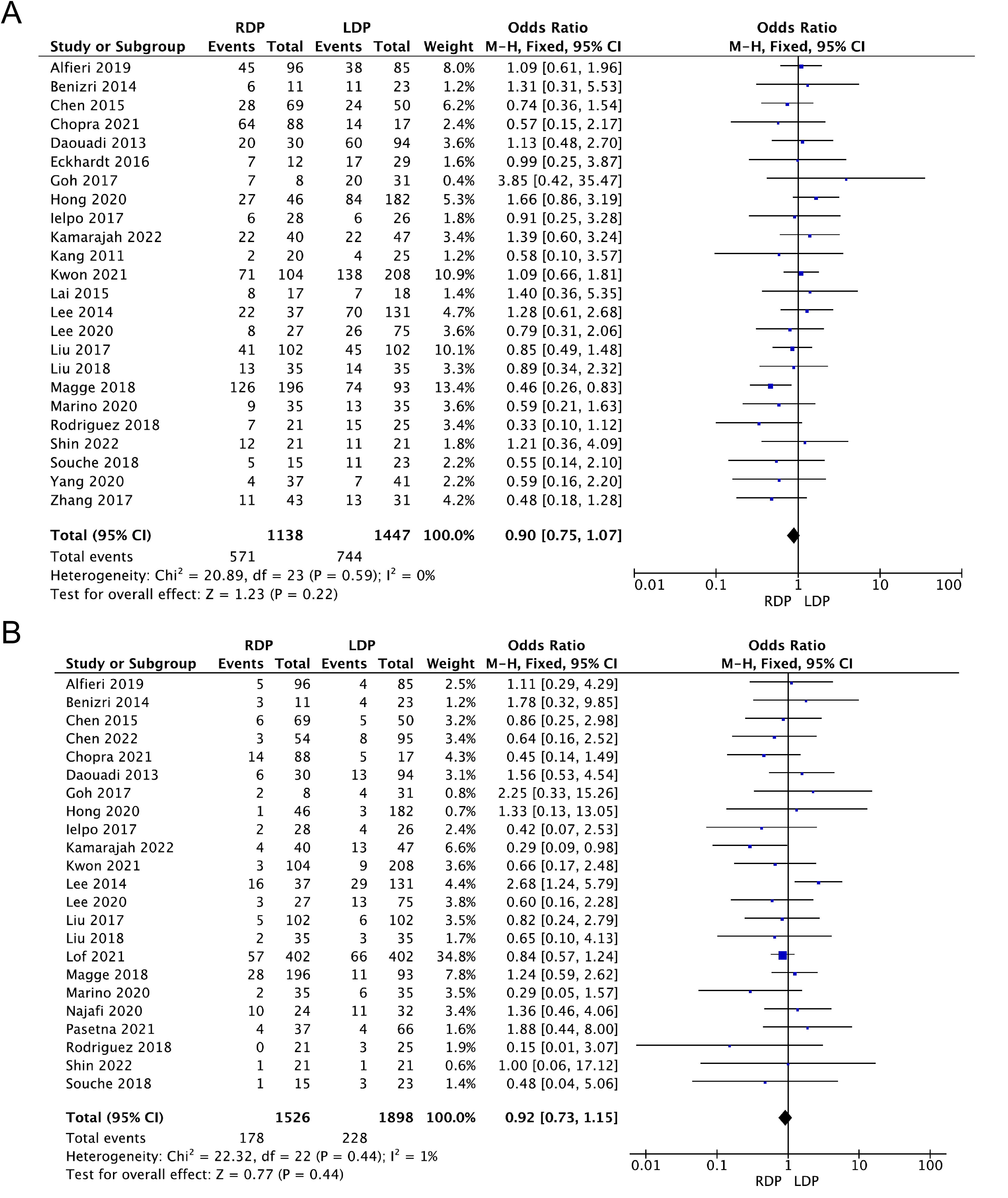

Supplement: Supplementary file 3 — S Fig. 3 Forest plot displaying the meta-analysis of overall complications (A) and major complications (B) (TIFF 814 KB) [file 13304_2022_1413_MOESM3_ESM.tiff]

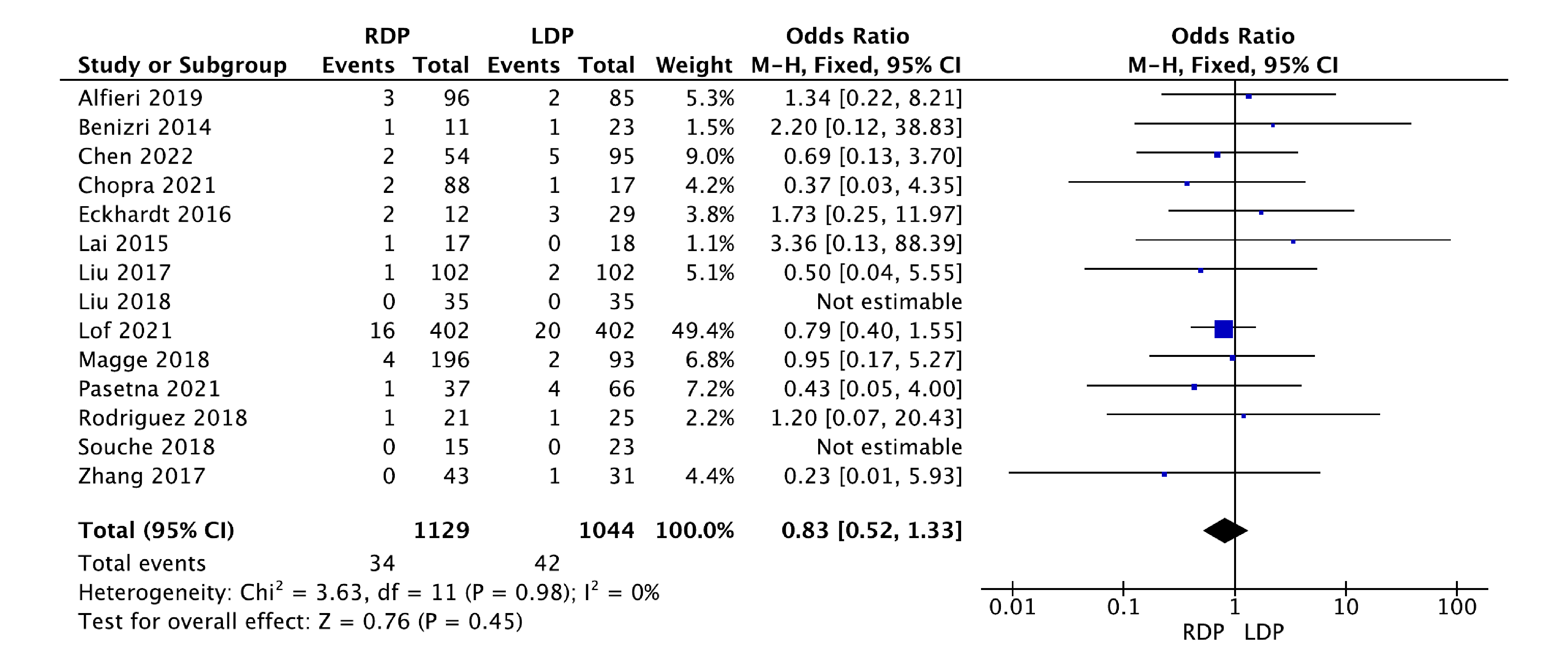

Supplement: Supplementary file 4 — S Fig. 4 Forest plot displaying the meta-analysis of postoperative haemorrhage (TIFF 276 KB) [file 13304_2022_1413_MOESM4_ESM.tiff]

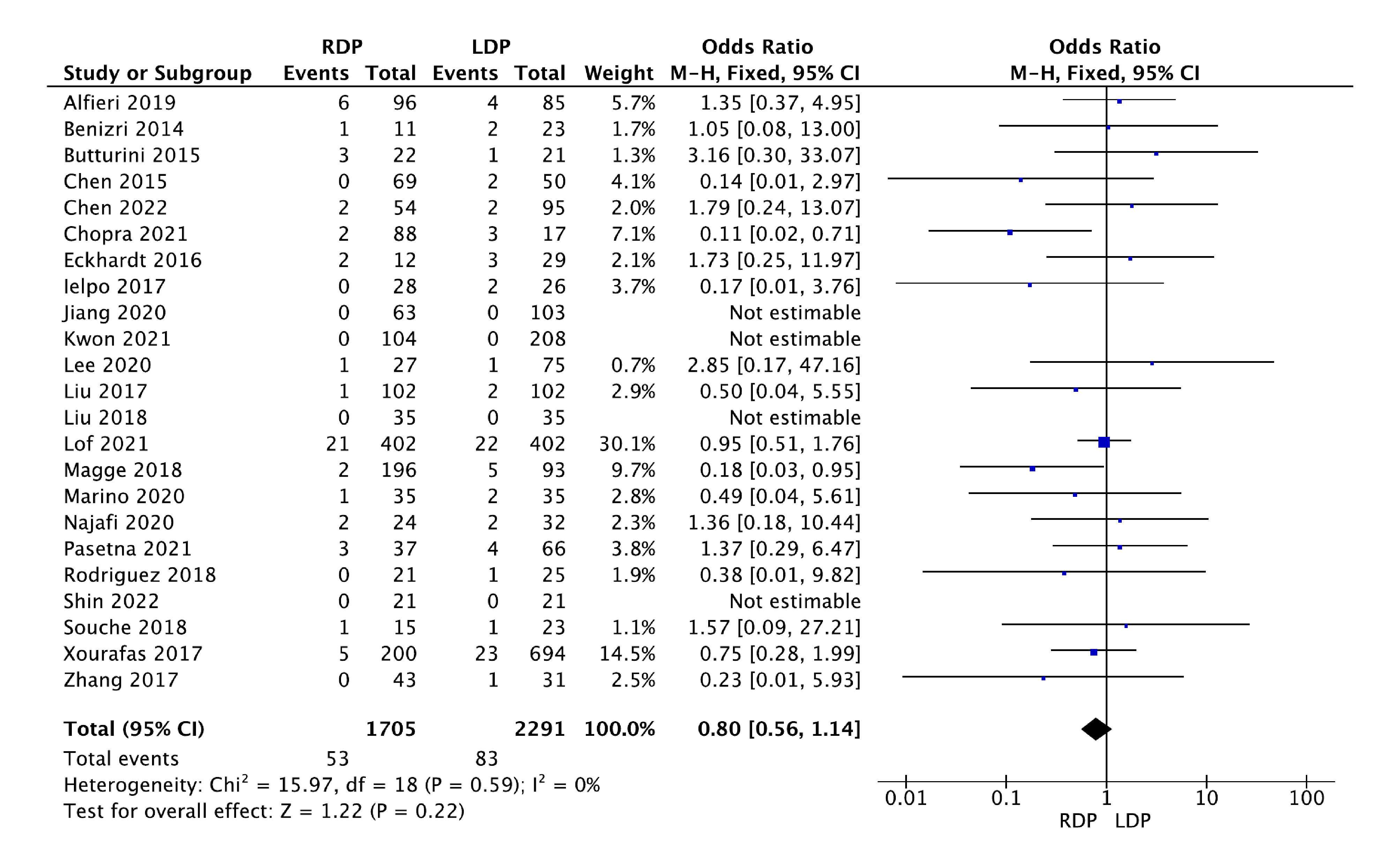

Supplement: Supplementary file 5 — S Fig. 5 Forest plot showing the meta-analysis of reoperation (TIFF 397 KB) [file 13304_2022_1413_MOESM5_ESM.tiff]

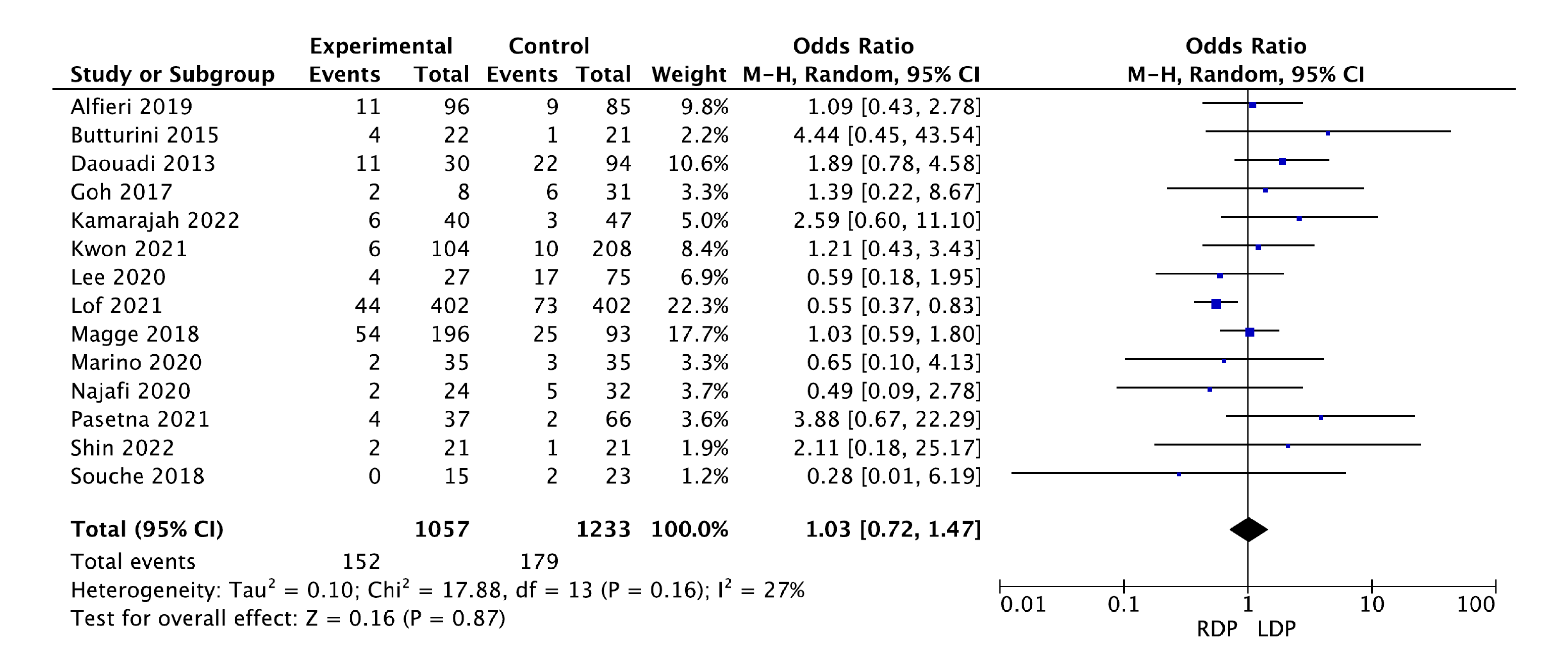

Supplement: Supplementary file 6 — S Fig. 6 Forest plot showing the meta-analysis of 90-day readmission (TIFF 289 KB) [file 13304_2022_1413_MOESM6_ESM.tiff]

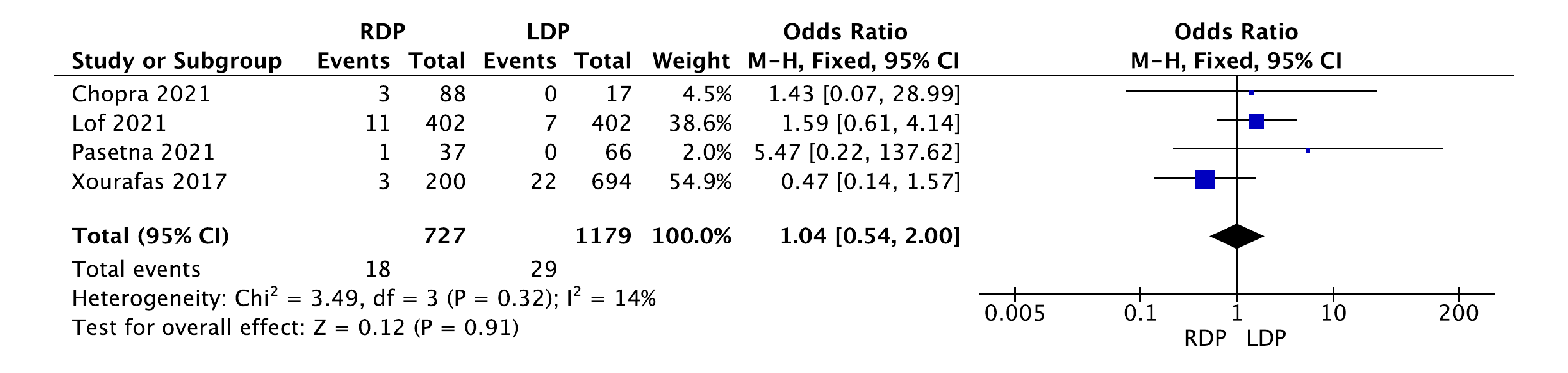

Supplement: Supplementary file 7 — S Fig. 7 Forest plot showing the meta-analysis of delayed gastric emptying (TIFF 151 KB) [file 13304_2022_1413_MOESM7_ESM.tiff]
