# Supplementary material for: Robotic versus laparoscopic distal pancreatectomy on perioperative outcomes: a systematic review and meta-analysis
Source: Updates Surg. 2022 Nov 15;75(1):7–21. doi: 10.1007/s13304-022-01413-3 (PMC9834369; doi:10.1007/s13304-022-01413-3)
Supplement: Supplementary file 8 — Supplementary file8 (DOCX 15 KB) [file 13304_2022_1413_MOESM8_ESM.docx]

Eegg’s test for publication bias of the included studies

| Measured Outcomes | No. Studies | Eegg's Test |  | Trim and filling | |
| --- | --- | --- | --- | --- | --- |
|  |  | Pr>\|t\|※ |  | P value(before) | Pvalue(after) |
| **Operative outcomes** |  |  |  |  |  |
| Operation time | 16 | 0.277 |  |  |  |
| Estimated blood loss (ml) | 7 | 0.206 |  |  |  |
| Intraoperative blood transfusion | 19 | 0.338 |  |  |  |
| Conversion to laparotomy | 29 | 0.310 |  |  |  |
| Spleen preservation | 12 | **0.010** |  | 0.000 | 0.000 |
| Kimura procedure | 11 | **0.009** |  | **0.002** | **0.184** |
| Number of lymph node dissected | 4 | 0.957 |  |  |  |
| R0 resection | 11 | 0.185 |  |  |  |
| **Postoperative outcomes** |  |  |  |  |  |
| Overall complication | 24 | 0.712 |  |  |  |
| Major complication | 23 | 0.410 |  |  |  |
| Clinical pancreatic fistula(grade B/C) | 30 | 0.418 |  |  |  |
| Delayed gastric emptying | 4 | 0.698 |  |  |  |
| Postoperative hemorrhage | 14 | 0.906 |  |  |  |
| Reoperation | 23 | 0.404 |  |  |  |
| Postoeprative hospital stay | 13 | 0.681 |  |  |  |
| 30-day mortality | 15 | 0.638 |  |  |  |
| 90-day mortality | 18 | 0.655 |  |  |  |
| Total cost | 5 | 0.117 |  |  |  |
| Operaion cost | 3 | 0.294 |  |  |  |

Major complications refer to the complications of grade >2 according to the Clavien-Dindo grade system. The Pancreatic fistula definition is according to the ISGPF criteria.
